# Methods and Applications of Social Media Monitoring of Mental Health During Disasters: Scoping Review

**DOI:** 10.2196/33058

**Published:** 2022-02-28

**Authors:** Samantha J Teague, Adrian B R Shatte, Emmelyn Weller, Matthew Fuller-Tyszkiewicz, Delyse M Hutchinson

**Affiliations:** 1 Centre for Social and Early Emotional Development School of Psychology Deakin University Geelong Australia; 2 Division of Tropical Health and Medicine, Department of Psychology College of Healthcare Sciences James Cook University Townsville Australia; 3 School of Engineering, Information Technology & Physical Sciences Federation University Melbourne Australia; 4 Murdoch Children’s Research Institute Melbourne Australia; 5 Centre for Adolescent Health, Royal Children’s Hospital Melbourne Australia; 6 Department of Paediatrics, University of Melbourne Melbourne Australia; 7 National Drug and Alcohol Research Centre, University of New South Wales Sydney Australia

**Keywords:** social media, SNS, mental health, disaster, big data, digital psychiatry

## Abstract

**Background:**

With the increasing frequency and magnitude of disasters internationally, there is growing research and clinical interest in the application of social media sites for disaster mental health surveillance. However, important questions remain regarding the extent to which unstructured social media data can be harnessed for clinically meaningful decision-making.

**Objective:**

This comprehensive scoping review synthesizes interdisciplinary literature with a particular focus on research methods and applications.

**Methods:**

A total of 6 health and computer science databases were searched for studies published before April 20, 2021, resulting in the identification of 47 studies. Included studies were published in peer-reviewed outlets and examined mental health during disasters or crises by using social media data.

**Results:**

Applications across 31 mental health issues were identified, which were grouped into the following three broader themes: estimating mental health burden, planning or evaluating interventions and policies, and knowledge discovery. Mental health assessments were completed by primarily using lexical dictionaries and human annotations. The analyses included a range of supervised and unsupervised machine learning, statistical modeling, and qualitative techniques. The overall reporting quality was poor, with key details such as the total number of users and data features often not being reported. Further, biases in sample selection and related limitations in generalizability were often overlooked.

**Conclusions:**

The application of social media monitoring has considerable potential for measuring mental health impacts on populations during disasters. Studies have primarily conceptualized mental health in broad terms, such as distress or negative affect, but greater focus is required on validating mental health assessments. There was little evidence for the clinical integration of social media–based disaster mental health monitoring, such as combining surveillance with social media–based interventions or developing and testing real-world disaster management tools. To address issues with study quality, a structured set of reporting guidelines is recommended to improve the methodological quality, replicability, and clinical relevance of future research on the social media monitoring of mental health during disasters.

## Introduction

Disaster mental health has emerged as a critical public health issue, with increasing rates of both disasters and mental health impacts on affected communities [1,2]. Disasters are natural (eg, earthquakes), technological (eg, industrial accidents), or human-caused events (eg, mass shootings) that have an acute and often unpredictable onset, are time delimited, and are experienced collectively [3]. The unexpected and evolving nature of disasters makes it challenging to monitor the population’s mental health in real time. Capturing current, accurate, and representative information about a population’s mental health during a disaster can assist in directing support to where it is most needed, monitoring the impact of response efforts, and enabling the delivery of targeted intervention. However, traditional methods of population-level mental health monitoring, such as large surveys of representative samples, can be logistically difficult to implement at short notice in an evolving and potentially deteriorating emergency context [4].

Research has investigated the potential benefits of social media (also known as social networking sites) data to capture the mental health status of affected population groups during a disaster, and monitor their recovery over time [5,6]. Social media data are advantageous because of their low cost of implementation; their ubiquity in the general population; the rich, real-time information that is shared by users (eg, photos, text, and video); and longitudinal assessment, which permits modeling of time trends and the temporal sequencing of target variables including events from the past [7]. Social media data also have particular strengths over traditional survey–based mental health monitoring [7,8]. This includes its ability to rapidly assess whole or specific populations to inform clinical decision-making, such as individuals in proximity to the disaster, those with pre-existing mental health conditions, or emergency responders. Further, social media data can support the real-time updating of mental health assessments, enabling administrators to identify and respond to shifting population needs, and transitions to new phases of the disaster event that may require a change in response strategy. Finally, social media data uniquely offer 2-way, synchronous communication opportunities, which can allow for rapid and scalable responses to misinformation, rumors, and stigma that may be harmful to mental health and the deployment of digital mental health support as required. However, the large quantity and unstructured nature of social media data also poses difficulties in terms of managing and extracting meaningful mental health information that is suitable for informing emergency response efforts.

A review capturing the strengths and weaknesses of the literature on disaster mental health monitoring via social media is both pertinent and timely, given the availability of social media analytic tools and the current COVID-19 crisis [9]. Previous reviews [10,11] have taken a narrower focus by examining the health literature; however, substantial research has been published in other interdisciplinary areas [12]. Notably, research from computer science and engineering is particularly relevant, and may offer sophisticated methodological advances to address challenges specific to large, unstructured data sets obtained from social media to indicate mental health outcomes [12,13]. This study aims to conduct a scoping review of the interdisciplinary literature assessing mental health in disasters using social media. Thus, this review aims to (1) identify how social media data have been applied to monitor mental health during disasters, including the type of social media and mental health factors that have been investigated; (2) evaluate the methods used to extract meaningful or actionable findings, including the mental health assessment, data collection, feature extraction, and analytic technique used; and (3) provide structured guidance for future work by identifying gaps in the literature and opportunities for improving methodologies and reporting quality.

## Methods

### Overview

A scoping review methodology was selected to map the key concepts, main sources, and types of evidence available in the literature on mental health using social media during disasters [14]. The review was performed adhering to the PRISMA-ScR (Preferred Reporting Items for Systematic Reviews and Meta-Analyses extension for Scoping Reviews) guidelines [15] and presents a subset of findings related to disaster mental health under a prospectively registered protocol (PROSPERO2020 CRD42020166421). The PRISMA-ScR Checklist is provided in Multimedia Appendix 1.

### Data Sources and Analysis

The following health and computer science databases were searched for relevant literature: PubMed or MEDLINE, PsycINFO, Cochrane Library, Web of Science, IEEE Xplore, and the ACM Digital Library. Details of the search strategy and variations of the key search terms can be found in Multimedia Appendix 2. Data were extracted using a standardized template adapted from similar reviews [10,12,13], which collated the following: (1) the aim and key findings of the research; (2) the disaster event details; (3) social media platform, data collection methods, and sample size; (4) area of mental health focus and assessment methods; and (5) analytic methods used, including preprocessing steps, feature extraction, and algorithm details. To analyze the data, a narrative review synthesis method was selected to best capture the methods and applications in the identified studies. A meta-analysis was not considered appropriate for this review given the broad range of mental health issues and analytic techniques used in the studies identified. As scoping reviews aim to provide an overview of the existing evidence regardless of methodological quality or risk of bias, no critical appraisal was performed [14,15]. However, missing information in articles was recorded in the data extraction template to assess overall methodological reporting quality.

### Search Strategy and Selection Criteria

A broad search strategy was adapted from the review of machine learning applications in mental health by Shatte et al [12]. Both health and information technology research databases were selected, including PubMed or MEDLINE, PsycINFO, Cochrane Library, Web of Science, IEEE Xplore, and the ACM Digital Library. Search terms were relevant to 3 themes—(1) mental health, (2) social media, and (3) big data analytic techniques—and the search was adapted to suit each database (Multimedia Appendix 2). The reference lists of all articles selected for review were manually searched for additional articles. The search was conducted on April 20, 2021, with no time or language delimiters.

The inclusion criteria were (1) articles that reported on a method or application of assessing mental health symptoms or disorders in a disaster, crisis, or emergency event; (2) articles that used social media data, with social media defined as any computer-mediated technology that facilitates social networks through user-generated content; (3) articles published in a peer-reviewed publication; and (4) articles available in English. Articles were excluded if they (1) did not report an original contribution to the research topic (eg, commentaries and reviews); (2) did not focus on a mental health application; (3) did not have full text available (eg, conference abstracts); and (4) solely used other internet-based activities, such as web browser search behaviors. Articles were screened by the lead author (SJT), with the second author (ABRS) blindly double-screening 5% of title and abstract articles and 10% of full-text articles, obtaining a 100% agreement rate.

## Results

### Overview of Article Characteristics

**Figure 1:**
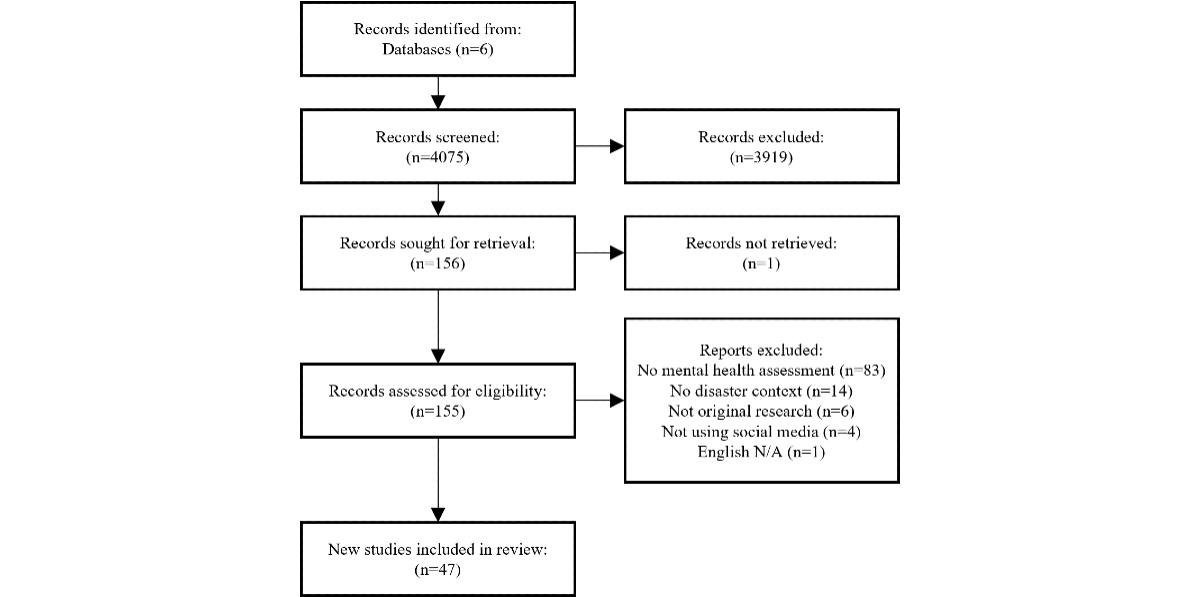
Preferred Reporting Items for Systematic reviews and Meta-Analyses extension for Scoping Reviews (PRISMA-ScR) Flowchart.

The search strategy identified 4075 articles, of which 47 were included in the review (Figure 1). The mean publication year was 2018 (*SD* 2.44 years), with the earliest article published in 2013. Health crises were the most commonly researched disaster events (24/47, 51%, including COVID-19, Middle East respiratory syndrome, and SARS) followed by human-made disasters (15/47, 32%, including terrorist attacks, school or mass shootings, technological and transportation accidents, and war), and natural disasters (12/47, 26%, including hurricanes, storms, floods, fires, earthquakes, tsunamis, and drought). Notably, the most commonly studied single disaster event was the COVID-19 pandemic (22/47, 47%), with human-made disasters being the most frequently studied disaster category before 2020. Disasters were reported most frequently in Asia (17/47, 36%), followed by North America (15/47, 32%), Europe (6/47, 13%), and South America (1/47, 2%). An additional 9 studies used social media data without any geographic restrictions. A total of 31 mental health issues were examined across the articles, with the most frequent being social media users’ affective responses (24/47, 51%), followed by anxiety (8/47, 17%), depression (7/47, 15%), stress (3/47, 4%), and suicide (3/47, 4%). The most common social media platform was Twitter (34/47, 72%), followed by Sina Weibo (6/47, 13%), Facebook (5/47, 11%), YouTube (4/47, 8%), Reddit (2/47, 4%), and other platforms (8/47, 17%). Overwhelmingly, the articles used an unobtrusive observational research design, with only 2 articles including any direct participation from users. Most articles reported the number of social media posts (44/47, 94%); in contrast, few studies reported the unique number of users included in the analysis (16/47, 34%). The mean number of posts in the included studies was 1,644,760.58 (SD 3,573,014.84, range 17-18,000,000), and the mean number of unique users was 164,318 (SD 250,791.27, range 49-826,961).

### Disaster Mental Health Applications

#### Overview

Three disaster mental-health application themes emerged: (1) estimating mental health burden (33/47, 70%; Table 1), which included articles that identified posts from the affected disaster region to track or predict changes to mental health over the disaster duration; (2) planning or evaluating interventions or policies (9/47, 19%; Table 2), which included articles that monitored mental health via social media as part of an intervention or policy evaluation; and (3) knowledge discovery (5/47, 11%; Table 3), which included a small number of articles that aimed to generate new insights into human behavior using social media in disaster contexts by developing theory and evaluating new hypotheses.

**Table 1:** Summary of articles estimating mental health burden from social media during a disaster.

Disaster category and reference			Disaster type		Disaster year		Disaster location		Mental health issue		Social media platform		Number of posts (number of users)		Analysis
**Natural disaster**															
	Gruebner et al [16]	Meteorological		2012		United States		Affective response		Twitter		344,957 (NR^a^)		GIS^b^ analysis	
	Gruebner et al [17]	Meteorological		2012		United States		Affective response		Twitter		1,018,140 (NR)		GIS analysis	
	Karmegam and Mappillairaju [18]	Hydrological		2015		India		Affective response		Twitter		5696 (NR)		Mixed effect model, spatial regression model, thematic analysis	
	Li et al [19]	Geophysical, biological		2009-2011		Japan, Haiti		Affective response		Twitter		50,000 (NR)		*t* test	
	Shekhar and Setty [20]	Geophysical, climatological, and hydrological		2015		Global		Affective response		Twitter		60,519 (NR)		Text mining; k-means clustering	
	Vo and Collier [21]	Geophysical		2011		Japan		Affective response		Twitter		70,725 (NR)		Naive Bayes, support vector machine, MaxEnt, J48, multinomial naive Bayes; Pearson correlation	
**Human-made disaster**															
	Doré et al [22]	Active shooter		2012-2013		United States		Affective response		Twitter		43,548 (NR)		Negative binomial regression	
	Glasgow et al [23]	Active shooter		2012-2013		United States		Grief		Twitter		460,000 (NR)		Multinomial naive Bayes; support vector machine	
	Gruebner et al [5]	Terrorist attack		2015		France		Affective response		Twitter		22,534 (NR)		GIS analysis	
	Jones et al [24]	Active shooter		2014-2015		United States		Affective response		Twitter		325,736 (6314)		Piecewise regression	
	Jones et al [25]	Terrorist attack		2015		United States		Affective response		Twitter		1,160,000 (25,894)		Time series, topic analysis	
	Khalid et al [26]	Terrorist attack		NR		NR		Trauma		Unspecified blogs and discussion boards		17 (NR)		Semantic mapping and knowledge pathways	
	Lin et al [27]	Terrorist attack		2015-2016		France, Belgium		Affective response		Twitter		18 Million (NR)		Multivariate regression analysis, survival analysis	
	Sadasivuni and Zhang [28]	Terrorist attack		2019		Sri Lanka		Depression		Twitter		51,462 (NR)		Gradient-based trend analysis methods, correlation, learning quotient, text mining	
	Saha and De Choudhury [29]	Active shooter		2012-2016		United States		Stress		Reddit		113,337 (NR)		Support vector machine classifier of stress, time-series analysis	
	Woo et al [30]	Accident		2011-2014		Korea		Suicide		Twitter		NR		Time-series analysis	
**Epidemic or pandemic**															
	Da and Yang [31]	Epidemic or pandemic		2020		China		Affective response		Sina Weibo		340,456 (NR)		Linear regression	
	Gupta and Agrawal [32]	Epidemic or pandemic		2020		India		Anxiety, depression, panic attacks, stress, suicide attempts		Twitter, Facebook, WhatsApp, and blogs		NR		Thematic analysis	
	Hung et al [33]	Epidemic or pandemic		2020		United States		Psychological stress		Twitter		1,001,380 (334,438)		Latent Dirichlet allocation	
	Koh and Liew [34]	Epidemic or pandemic		2020		Global		Loneliness		Twitter		NR (4492)		Hierarchical clustering	
	Kumar and Chinnalagu [35]	Epidemic or pandemic		2020		NR		Affective response		Twitter, Facebook, YouTube, and blogs		80,689 (NR)		Sentiment analysis bidirectional long short-term memory	
	Lee et al [36]	Epidemic or pandemic		2020		Japan, Korea		Affective response		Twitter		4,951,289 (NR)		Trend analysis	
	Li et al [37]	Epidemic or pandemic		2020		China		Anxiety, depression, indignation, and Oxford happiness		Sina Weibo		NR (17,865)		*t* test	
	Low et al [38]	Epidemic or pandemic		2018-2020		Global		Eating disorder, addiction, alcoholism, ADHD^c^, anxiety, autism, bipolar disorder, BPD^d^, depression, health anxiety, loneliness, PTSD^e^, schizophrenia, social anxiety, suicide, broad mental health, COVID-19 support		Reddit		NR (826,961)		Support vector machine, tree ensemble, stochastic gradient descent, linear regression, spectral clustering, latent Dirichlet allocation	
	Mathur et al [39]	Epidemic or pandemic		2019-2020		Global		Affective response		Twitter		30,000 (NR)		Sentiment analysis	
	Oyebode et al [40]	Epidemic or pandemic		2020		Global		General mental health concerns		Twitter, YouTube, Facebook, Archinect, LiveScience, and PushSquare		8,021,341 (NR)		Thematic analysis	
	Pellert et al [41]	Epidemic or pandemic		2020		Austria		Affective response		Twitter and unspecified chat platform for students		2,159,422 (594,500)		Trend analysis	
	Pran et al [42]	Epidemic or pandemic		2020		Bangladesh		Affective response		Facebook		1120 (NR)		Convolutional neural network and long short-term memory	
	Sadasivuni and Zhang [43]	Epidemic or pandemic		2020		Global		Depression		Twitter		318,847 (NR)		Autoregressive integrated moving average model	
	Song et al [44]	Epidemic or pandemic		2015		South Korea		Anxiety		Twitter, Unspecified blogs and discussion boards		8,671,695 (NR)		Multilevel analysis, association analysis	
	Xu et al [45]	Epidemic or pandemic		2019-2020		China		Affective response		Sina Weibo		10,159 (8703)		Content analysis, regression	
	Xue et al [46]	Epidemic or pandemic		2020		Global		Affective response		Twitter		4,196,020 (NR)		Latent Dirichlet allocation, sentiment analysis	
	Zhang et al [47]	Epidemic or pandemic		2020		United States		Depression, anxiety		YouTube		294,294 (49)		Regression, correlation, feature vector	

^a^NR: not reported.

^b^GIS: geographic information system.

^c^ADHD: attention-deficit/hyperactivity disorder.

^d^BPD: borderline personality disorder.

^e^PTSD: posttraumatic stress disorder.

**Table 2:** Summary of articles planning or evaluating interventions or policies from social media during a disaster.

Disaster category and reference			Disaster type		Disaster year		Disaster location		Mental health issue		Social media platform		Number of posts (number of users)		Analysis
**Natural disaster**															
	Baek et al [48]	Geophysical, accident		2011		Japan		Anxiety		Twitter		179,431 (NR^a^)		Time-series analysis	
**Human-made disaster**															
	Budenz et al [49]	Active shooter		2017		United States		Mental illness stigma		Twitter		38,634 (16,920)		Logistic regression	
	Glasgow et al [50]	Active shooter		2011-2012		United States		Coping and social support		Twitter		NR		Classifier (unspecified), qualitative coding analysis	
	Jones et al [6]	Active shooter		NR		United States		Psychological distress		Twitter		7824 (2515)		Time-series analysis	
**Epidemic or pandemic**															
	Abd-Alrazaq et al [51]	Epidemic or pandemic		2020		Global		Affective response		Twitter		167,073 (160,829)		Latent Dirichlet allocation	
	He et al [52]	Epidemic or pandemic		2020		Americas and Europe		Depression, mood instability		YouTube		255 (NR)		Touchpoint needs analysis	
	Massaad and Cherfan [53]	Epidemic or pandemic		2020		Undisclosed		Service access/needs		Twitter		41,329 (NR)		Generalized linear regression, k-means clustering	
	Wang et al [54]	Epidemic or pandemic		2020		China		Subjective well-being		Sina Weibo		NR (5370)		Regression, analysis of variance	
	Zhou et al [55]	Epidemic or pandemic		2020		China		Affective response		Sina Weibo		8,985,221 (NR)		Latent Dirichlet allocation	

^a^NR: not reported.

**Table 3:** Summary of articles discovering new knowledge and generating hypotheses from social media during disasters.

Disaster category and reference			Disaster type		Disaster year		Disaster location		Mental health issue		Social media platform		Number of posts (number of users)		Analysis
**Natural disaster**															
	Gaspar et al [56]	Biological		2011		Germany		Coping		Twitter		885 (NR^a^)		Qualitative coding analysis	
	Shibuya and Tanaka [57]	Geophysical		2011		Japan		Anxiety		Facebook		873,005 (16,540)		Hierarchical clustering	
**Human-made disaster**															
	De Choudhury et al [58]	War		2010-2012		Mexico		Anxiety, PTSD^b^ symptomatology, affective response		Twitter		3,119,037 (219,968)		Pearson correlation, *t* test	
**Epidemic or pandemic**															
	Van Lent et al [59]	Epidemic or pandemic		2014		Netherlands		Affective response		Twitter		4500 (NR)		Time-series analysis	
	Ye et al [60]	Epidemic or pandemic		2020		China		Prosociality, affective response		Sina Weibo		569,846 (387,730)		Regression	

^a^NR: not reported.

^b^PTSD: posttraumatic stress disorder.

#### Estimating Mental Health Burden

Articles that estimated the mental health burden after a disaster typically examined the presence of any negative affect in posts using sentiment or affect dictionaries over the duration of the disaster. For example, Gruebner et al [16] monitored the mental health of New Yorkers during the Hurricane Sandy disaster of 2012 using sentiment analysis of Twitter posts. Over 11 days surrounding the hurricane’s landfall, 24 spatial clusters of basic emotions were identified: before the disaster, clusters of anger, confusion, disgust, and fear were present; a cluster of surprise was identified during the disaster; and finally a cluster of sadness emerged after the disaster. Expanding on this, Jones et al [24] examined the mental health trauma impact of school shooting events across 3 US college campuses using a quasi-experimental design. Specifically, an interrupted time series design was used with a control group and a reversal when the next shooting event occurred in the original control group’s college. Increased negative emotion was observed after all 3 shooting events, particularly among users connected to the affected college campus within 2 weeks of the shooting. Finally, a few articles explored specific mental health conditions rather than general negative sentiments (eg, depression [28], stress [29], and anxiety [30]). One notable study by Low et al [38] examined mental health during the impact of the initial stages of the COVID-19 pandemic on 15 mental health support groups on Reddit, allowing for disorder-specific monitoring and comparison. An increase in health anxiety and suicidality was detected across all mental health communities. In addition, the attention deficit hyperactivity disorder, eating disorder, and anxiety subreddits experienced the largest change in negative sentiment over the duration of the study and became more homogeneous to the health anxiety subreddit over time.

#### Planning or Evaluating Interventions or Policies

Articles evaluating the mental health impact of disaster interventions or policies focused primarily on the association between public health measures during the COVID-19 crisis, such as lockdowns, personal hygiene, and social distancing, with social media users’ mental health. For example, Wang et al [54] compared the subjective well-being of Sina Weibo users in China during lockdown versus those who were not, finding that lockdown policy was associated with an improvement in subjective well-being, following very low initial levels recorded earlier in the pandemic. Next, 2 studies examined the relationship between crisis communication and the mental health of social media users impacted by a disaster, including government communication during the 2011 Great East Japan Earthquake or Fukushima Daiichi Nuclear Disaster and a school shooting event in the United States [6,48]. Both studies found that unclear or inconsistent official communication delivered via social media was associated with a proliferation of rumors and public anxiety. Finally, 3 studies examined the mental health service needs of social media users following disasters, including telehealth needs during the COVID-19 pandemic, mental illness stigma following a mass shooting, and the support offered to disaster victims after a tornado and mass shooting [49,50,53]. Combined, these studies identified potential methods to assess the need for policies or interventions for mental health issues following a disaster by examining the access and availability of services to social media users.

#### Knowledge Discovery

The 5 articles that were classified as knowledge discovery aimed to evaluate theories of human behavior and mental health during disasters using social media data. This included examining the impact of psychological distance on the attention a disaster receives from social media users [59], prosocial behavior, coping, and desensitization to trauma during the disaster [56,58,60] and predicting recovery from social media users’ purchasing behaviors and intentions [57].

### Disaster Mental Health Methods

#### Assessing Mental Health

A total of 4 methods were identified to assess mental health using social media data. First, linguistic methods were the most frequently used (26/47, 55%), such as the presence of keywords generated by the study authors (eg, *loneliness* and synonyms [34]) applying established dictionaries (eg, the Linguistic Inquiry and Word Count (LIWC) dictionary [27]), or pretrained language models (eg, Sentiment Knowledge Enhanced Pre-training [31]). Second, human assessment was used in 18 studies, with 61% (11/18) using human annotators to conduct qualitative coding, typically for nuanced mental health information (eg, type of social support received [50]), and 39% (7/18) of studies interpreting a mental health topic from a topic modeling analysis. Next, 2 studies used mental health forum membership to indicate mental health problems [29,38], with Saha and De Choudhury [29] using a novel method of transfer learning from a classifier trained on a mental health subreddit (r/stress) and a random sample of Reddit posts to identify posts with high stress on college-specific subreddits following gun-related violence on campus. Finally, mental health questionnaires were used in 2 studies, specifically, the Patient Health Questionnaire-9 item and 7-item Generalized Anxiety Disorder scale [47], and Psychological Wellbeing scale [54].

Very few studies had implemented methods to improve data quality and validity of mental health assessments. A total of 3 studies included direct coding of posts using validated mental health measures. For example, Saha et al [29] directly annotated r/stress Reddit posts for high or low stress using the Perceived Stress Scale to develop a classifier. Gaspar et al [56] directly coded 885 tweets for coping during a food contamination crisis in Germany with a coping classification framework by Skinner et al [61]. Furthermore, very few studies had included a control or comparison group to delineate the relationship between the disaster and mental health. Comparison groups were identified in 8 studies: 63% (5/8) studies used a between-groups design typically selecting users from a different location to the disaster event as a comparison [23,31,38,49,54], and 38% (3/8) used a within-subjects comparison by comparing social media users against their own data from a different point in time [27-29].

#### Data Collection and Preprocessing

Data were primarily collected via the platform’s public streaming application programming interface (API) (20/47, 42%); for example, the Twitter representational state transfer (REST) API. Digital archives and aggregation services of social media data were the next most commonly used method (10/47, 21%), such as the Harvard Center for Geographic Analysis Geotweet Archive [5,17] and the TwiNL archive [59]. Finally, a few studies used other techniques, including web crawlers (8/47, 17%), third-party companies (2/47, 4%), and other novel methods (2/47, 4%) such as having participants download and share their use patterns via Google Takeout [47]. A handful of studies did not report their data collection method (6/47, 13%). In most studies (39/47, 83%), individual posts were the unit of analysis, rather than the user contributing to those posts (8/47, 17%). Sampling methods typically involved selecting posts or users with location data and key terms related to crisis events. Identifying the location of users or posts included extracting location data from profile pages [57], selecting users or posts with geotagged posts [53], or identifying users or posts with hashtags of the crisis location [58]. A few studies selected users that followed organizations local to the crisis event, including college campus subreddits or Twitter profiles [24,29]. Key terms related to the crisis event included hashtags or key terms of the event (eg, #Ebola [59]) or mental health keywords (eg, suicide or depression synonyms [30]).

Preprocessing steps to prepare the data for analysis were reported in 36 articles, and typically involved translating posts into a single language (typically English) or removing posts in other languages, identifying and removing posts containing advertising or spam, and removing duplicate posts (eg, retweets). Usernames, URLs, and hashtags were either removed or normalized. Many studies have also removed posts without keywords related to crisis events, such as location or crisis-specific terms. Studies using natural language processing methods have conducted additional preprocessing steps to clean the data before machine analysis, including removing punctuation, stopwords, and nonprintable characters, and stemming, lemmatizing, and tokenizing words. No studies reported how missing data were handled, or evidence of strategies to improve data quality, such as minimum thresholds of engagement with social media or mental health disclosure.

#### Feature Extraction

Several features were extracted across studies, which have been grouped into linguistic, psycholinguistic, demographic, and behavioral features. Psycholinguistic features were the most frequently identified (34/47, 72%), and included sentiment (positive, negative, or neutral), affect (positive or negative), time orientation (past, present, or future tense), personal concerns (eg, LIWC’s work, money, and death dictionaries), humor, valence, arousal, and dominance. These features were typically extracted using established lexicons, such as Stanford CoreNLP and LIWC. Some studies used direct coding of posts for psycholinguistic features, for example, attitudes toward vaccinations [62], and whether the post contained fear for self or others [59]. Linguistic features were present in 24 studies and included n-grams, term frequency–inverse document frequency statistics, bag of words, usernames, hashtags, URLs, and grammar and syntax features. Demographic features were less commonly used (12/47, 26%), but typically involved the location associated with the social media post. Some studies engineered location data into new metrics, for example, negative emotion rate per local area surrounding the disaster [18]. A few studies identified the age and gender of users by extracting the information from user profiles [37] or via age and gender lexicons (eg, Genderize API [27]).

Although most studies used linguistic, psycholinguistic, and demographic features only, a few studies also extracted additional features about users’ behaviors. Behavioral features were identified in 7 studies and included metrics such as a user’s social media post rate [22], sharing of news site URLs [27], and directly coded behaviors such as handwashing and social distancing [44]. Behavioral features also included the aspects of the user’ social network (identified in 3 studies), including the user’s friend and follower counts [53,63], and social interaction features (identified in 4 studies), such as the use of @mentions [27,63] and retweets [6].

#### Analytic Methods

A range of analytic methods have been used across studies, including machine learning, statistical modeling, and qualitative techniques. The most common approach was trend analysis to examine temporal changes in mental health before, during, and/or after a disaster event (24/47, 51%). Geospatial analytic methods were also identified (4/47, 8%), including geographic information system methods to examine location-based differences in mental health factors during disasters [18]. A few studies have used machine learning classifiers to categorize posts or users into groups (eg, high vs low stress in study by Saha et al [29]), namely support vector machine (6/47, 13%), maximum entropy classifier (1/47, 2%), multinomial naïve Bayes (2/47, 4%), long short-term memory (2/47, 4%), naive Bayes (1/47, 2%), J48 (1/47, 2%), convolutional neural network (1/47, 2%), tree ensemble (1/47, 2%), and stochastic gradient descent classifier (1/47, 2%). Validation metrics for these studies involved train-test splits and k-folds cross-validation, with k ranging from 5 to 10. Finally, a number of studies implemented topic modeling (12/47, 25%) and qualitative analytic approaches (8/47, 17%) to identify themes in social media discussions during the disaster. Topic modeling included latent Dirichlet allocation (5/12, 42%) (eg, [33]), k-means clustering (3/12, 25%) (eg, [20]), hierarchical cluster analysis (2/12, 17%) (eg, [34]), and other clustering methods (3/12, 25%) (eg, [19]). Qualitative analyses included topic analysis (1/8, 12%) [25], thematic analysis (2/8, 25%) (eg, [64]), touchpoint needs analysis (1/8, 12%) [52], content analysis (1/8, 12%) [45], and other coding techniques (3/8, 37%) (eg, [26]).

#### Ethical Considerations

There was limited discussion of the ethics of pervasive mental health monitoring in the identified studies (18/47, 38%). A total of 7 studies noted that their research protocol was reviewed and approved by an institutional review board, and 5 stated that their research was exempt from ethical review. A total of 8 studies noted participant privacy concerns, addressing this by anonymizing usernames or handles, accessing only public information about users, and not publishing verbatim quotes from posts. Furthermore, 5 articles provided information capable of reidentifying social media users involved in their study, such as direct quotes from users and social media post IDs. In addition, 2 studies noted that their work complied with the platform’s data use policy. Only 1 study sought direct consent from social media users to access their data.

#### Methodological Reporting

As noted previously, the included literature was inconsistent in its reporting of key factors, such as the place, date, and type of the disaster; social media platform used; number of unique users within the data set; handling of missing data; preprocessing steps; data quality requirements; final feature set included in the analysis; rationale for mental health assessment methods, analysis plan, overall study design; and ethical considerations. These details are important for accuracy in the interpretation and reproduction of research findings. To assist researchers in improving their study quality and reporting, Textbox 1 presents a checklist of key methodological decisions to be considered and reported, where appropriate. The checklist was based on the data extraction template used for the current review and is intended as a guide for key study design and reporting considerations. None of the included studies met all of the reporting criteria.

**Reporting checklist for the social media analysis of mental health during disasters.**

**Research design and theoretical formulation**

- Type, place, and dates of disaster event
- Use and selection of control or comparison group (eg, between or within subject design with comparison users at a different time point, geographic location, and social media platform)
- Consideration of causal inference methods (eg, natural experiment design, positive and negative controls)
- Theoretical justification of research question (eg, theories of mental health onset or progression and web-based social interaction)

**Data collection**

- Social media platforms targeted (eg, Twitter, Reddit)
- Data collection method specified (eg, application programming interface, scraping, and data provided by user directly)
- Sampling frame restrictions (eg, dates, geolocation, keyword requirements)
- Number of unique social media posts and users
- Consideration of sampling biases and confounding factors (eg, matching demographics to census data)

**Mental health assessment**

- Methods for assessing mental health status (eg, sentiment analysis, human annotation)
- How ground truth was obtained (eg, manual coding of social media posts, participant completion of validated psychological measures)
- Clinical justification for assessment method (ie, evidence to support the clinical validity and reliability of the assessment method)

**Preprocessing and feature extraction**

- Details of any manipulations to the data (eg, text translation)
- Criteria for removed social media posts or users (eg, spam or advertisements)
- Use of minimum engagement thresholds (eg, users were required to have >1 post per week)
- Handling of missing data (eg, multiple imputation)
- Transformation of data (eg, tf-idf or word to vector)
- Explicit number of features extracted and used in analysis

**Analysis**

- Analytic technique or algorithm selection justification (ie, techniques suitability to address the research question)
- Consideration of statistical techniques for causal inference (eg, propensity scores)
- Validation technique and metrics (eg, k-fold cross-validation; test or train split)
- Performance or fit of algorithm (eg, F1-score, accuracy)
- Number of data points
- Error analysis and explanation (eg, sensitivity analysis)
- Feature reduction techniques (eg, principal-component analysis, forward feature selection)

**Ethical considerations**

- Compliance with social media platform’s data policy (eg, Twitter terms of use)
- Consideration of ethical research obligations (eg, ethical review board approval)
- Minimizing human exposure to participant data where possible (eg, restricting human researcher access to data when not required, use of machine-based analyses)
- Individual participant data not reported without consent (ie, aggregate results reported only)
- Use of public social media data only (ie, no login or interaction with users required to access the data)
- Anonymization of data to maintain participant privacy (eg, removing usernames or identifiable photos, natural language processing techniques such as named entity recognition, publishing metadata only in data repositories)

## Discussion

### Principal Findings

This study synthesized the literature assessing mental health in disasters using social media, highlighting current applications and methods. Research has predominantly focused on retrospectively monitoring the negative affect of social media users local to a disaster area using established psycholinguistic dictionaries. Emerging research has assessed other public mental health issues, including the impact of news and government messaging, telehealth access, and mental health stigma. Analytic techniques are sophisticated in identifying relevant social media users and modeling their changing mental health after a disaster. Overall, social media offers a promising avenue to efficiently monitor public mental health during disasters and is capable of overcoming many logistical challenges of traditional methods such as large sample sizes, before, during, or after event data, and collection of comparison or control data.

As an emerging field, there are understandably significant gaps for future research to address. It is evident that mental health is broadly conceptualized by researchers as negative affect or distress, assessed using sentiment or affect dictionaries. However, it remains unclear whether spikes in posts with negative affect detected during and after disasters are clinically meaningful changes warranting intervention or the natural course of psychopathology following a distressing event [65]. More participatory research could address this issue by combining passive social media monitoring with validated psychological measures capable of capturing both distress and dysfunction in the affected population [47]. Furthermore, few studies have examined the use of social media to assess the impact of disasters on the mental health of vulnerable populations, including those with pre-existing mental health issues. Only one identified study compared how different mental health communities were affected following a disaster [38], finding both similarities and differences in responses between disorder-specific communities. Researchers may consider investigating how specific mental health conditions can be detected and monitored using social media, particularly focusing on disorders that are likely to experience exacerbated symptomatology during disasters.

Beyond community mental health assessment, it is also clear that there is scope to improve the field by using more robust research methods. Most studies used an observational pre-post crisis event design, with few studies including a control or comparison group or causal inference methods. Social media data offer many opportunities for rich study designs, including natural experiments or positive and negative control designs. Such designs would assist in delineating the impact of crisis events and response efforts on mental health. Researchers should consider how to collect representative samples or control for demographic differences in analyses, for example, by matching sample data with census information, with very few studies considering the generalizability of results. Notably, researchers in this field should be mindful of differences between measures of association (eg, *lockdown measures were associated with increased loneliness on Twitter)* and causal claims (eg, *the terrorist attack increased anxiety on Reddit*) in observational studies. Causal claims should not be inferred outside of causal models, as there may be multiple possible causes to the observed effects that are not able to be measured and controlled in observational research designs. Importantly, controlled studies may be infeasible when modeling unforeseen events such as disasters or large, varied populations over time; therefore, finding associations through public health monitoring could be incredibly useful.

Further, few studies have considered the ethical implications of their research. Current ethical guidelines state that the large-scale and public nature of social media data may enable such research to be exempt from review by ethics committees. Nevertheless, researchers need to be mindful of the sensitive nature of inferring mental health states using unvalidated methods from social media data that may not be anonymous. The community needs to develop protocols for managing social media users’ privacy while maintaining high-quality research practices, including balancing participant privacy with open science principles of data sharing [66]. Researchers should be mindful that, despite the public nature of the collected data, social media users may have privacy concerns about their data being aggregated into a permanent, curated data set to enable inferences about their mental health, without their knowledge or consent [67]. Ethical data sharing protocols could include sharing the data with qualified researchers to improve reproducibility and open science practices and removing ties between a user’s posts and profile in publications, such as paraphrasing quotes and publishing meta data rather than identifiable profiles or posts. Researchers also need to ensure that their study complies with the data use policies of the platforms they are accessing, including user privacy requirements and the platform’s preferred data access methods.

Finally, there are exciting avenues for future research that will greatly progress the field. Emerging research has developed and evaluated new theories and hypotheses of mental health during disasters using social media data, providing exciting advances in our understanding of how social media data can be capitalized for knowledge discovery [58,60]. Such research should be encouraged by both computer and mental health scientists, given that other public health applications can be achieved by government organizations as part of their disaster response efforts [65]. No study has evaluated the clinical utility of social media mental health monitoring by developing and testing real-world disaster management tools. Creating simple tools for disaster mental health monitoring and translating them into real-world settings will likely elicit new challenges that may not be present in the laboratory, particularly when applied across different clinical and emergency contexts. Another promising avenue for future research is to combine social media mental health monitoring with interventions. This could be achieved by detecting individuals in need of support and directing them toward available interventions, including those designed for disaster contexts (eg, psychological first aid or debriefing and crisis counseling), tailored mobile health and eHealth tools, or broader social media–based interventions [68]. Finally, the field would greatly benefit from more collaboration between mental health and computer science experts to bring nuance to the conceptualization of mental health and its assessment alongside sophisticated analytic methods.

There are 2 key limitations to this review that should be considered along with the study findings. First, the scoping review methodology entails a rapid and broad search to identify and map relevant literature. To balance these requirements, the search strategy used broad search terms and excluded non-English and non–peer reviewed literature [69]. A more in-depth review would potentially capture additional relevant studies, but would be less feasible to complete and would date quickly given the rapidly evolving nature of the field. Second, this study did not delineate how effectively social media can be used to capture mental health impact during a disaster event, as validation of mental health assessments against other measures was limited. To address both limitations, future work could conduct an in-depth review of specific mental health issues, social media, or disaster contexts, guided by the framework developed in this study.

### Conclusions

In conclusion, there have been exciting advances in research aimed at monitoring mental health during disasters using social media. Overall, social media data can be harnessed to infer mental health information useful for disaster contexts, including negative affect, anxiety, stress, suicide, grief, coping, mental illness stigma, and service access. Sophisticated analytic methods can be deployed to extract features from social media data and model their geospatial and temporal distribution over the duration of the crisis event. As an emerging field, there are substantial opportunities for further work to improve mental health assessment methods, examine specific mental health conditions, and trial tools in real-world settings. Combined, such platforms may offer a useful avenue for monitoring mental health in contexts where formal assessments are difficult to deploy and may potentially be harnessed for response effort monitoring and intervention delivery.

## Appendix

Multimedia Appendix 1 PRISMA (Preferred Reporting Items for Systematic Reviews and Meta-Analyses) extension for Scoping Reviews checklist.

Multimedia Appendix 2 Search details.

## Abbreviations

API: application programming interface
LIWC: Linguistic Inquiry and Word Count
PRISMA-ScR: Preferred Reporting Items for Systematic Reviews and Meta-Analyses extension for Scoping Reviews
