# Supplementary material for: Methods and Applications of Social Media Monitoring of Mental Health During Disasters: Scoping Review
Source: JMIR Ment Health. 2022 Feb 28;9(2):e33058. doi: 10.2196/33058 (PMC8922153; doi:10.2196/33058)
Supplement: Multimedia Appendix 2 [file mental_v9i2e33058_app2.docx]

# Multimedia Appendix 2: Search strategy

Search details

Restrictions: English papers published in a peer-reviewed journal or conference proceeding.

Medical Subject Headings (MeSH)

1. Anxiety OR agoraphobia OR phobia* OR panic OR “posttraumatic stress” OR “mental health” OR “mental illness*” OR depress* OR “affective disorder*” OR bipolar OR “mood disorder*” OR psychosis OR psychotic OR schizophre* OR well-being OR wellbeing OR “quality of life” OR self-harm or “self-injury” OR stress* OR distress* OR mood OR “body image” OR “eating disorder*”

2. “Social Media”, OR “Social Network” OR “Social Networking Sites” OR “web blog” OR newsgroup OR blog OR forum OR Twitter OR WhatsApp OR Facebook OR Weibo OR Snapchat OR YouTube OR Instagram OR MySpace OR Orkut OR WeChat OR Google+ OR Neverblog OR QQ OR Viber

3. “Machine Learning” OR “Data Mining” OR “Big Data” OR “Text Analysis” OR “Text Mining” OR “Predictive Analytics” OR “Deep Learning” OR “Natural Language Processing” OR “Artificial Intelligence”

Note: Future work should also include “post-traumatic stress” or “PTSD” as well as “posttraumatic stress”
